# Moral Resilience and Its Associations with Workplace Gaslighting and Quiet Quitting: A Cross-Sectional Study of White-Collar Workers in Greece

**DOI:** 10.3390/healthcare14162552

**Published:** 2026-08-14

**Authors:** Ourania-Themis Kouvela, Nikolaos Veskoukis, George Kritsotakis, Michael Igoumenidis

**Affiliations:** 1 Department of Nursing, School of Health Rehabilitation Sciences, University of Patras, 26334 Patras, Greece; up1105857@upatras.gr (O.-T.K.); up1104401@upatras.gr (N.V.); 2 Department of Business Administration & Tourism, Hellenic Mediterranean University, 71410 Heraklion, Greece; gkrits@hmu.gr

**Keywords:** moral resilience, workplace gaslighting, quiet quitting, white-collar workers, public sector employees, employee wellbeing, cross-sectional study

## Abstract

**Highlights:**

**What are the main findings?**

- Quiet quitting and supervisory gaslighting were both reported in a Greek white-collar workforce, with lack of motivation and abuse of power as the most pronounced components.
- Moral resilience was independently associated with lower quiet quitting and lower gaslighting.

**What are the implications of the main findings?**

- Strengthening moral resilience may help white-collar employees who face disengagement and manipulative supervision, although longitudinal evidence is needed.
- Reducing quiet quitting calls for attention to supervisory behavior and the ethical quality of the work environment.

**Abstract:**

**Background/Objectives:** Quiet quitting, workplace gaslighting, and moral resilience are increasingly recognized as important to employee wellbeing, yet they have rarely been studied together, and evidence from white-collar workforces outside healthcare is scarce. This study examined these three constructs, as well as the associations between them, among white-collar employees of a governmental organization in Greece. **Methods:** A cross-sectional study was conducted with a self-report questionnaire completed by 433 employees, about 90% of the target population. Quiet quitting, workplace gaslighting, and moral resilience were measured with the Quiet Quitting Scale, the Gaslighting at Work Scale, and the revised Rushton Moral Resilience Scale (RMRS-16). **Results:** The mean quiet quitting score was 2.26 (SD 0.69), the mean gaslighting score was 1.84 (SD 0.76), and the mean moral resilience score was 3.08 (SD 0.43). In multivariable analysis, higher moral resilience was independently associated with lower quiet quitting (b = −0.52, 95% CI: −0.65 to −0.39) and lower gaslighting (b = −0.49, 95% CI: −0.65 to −0.34). Higher gaslighting was associated with higher quiet quitting (b = 0.27, 95% CI: 0.19 to 0.35). Men and younger employees reported higher levels of both. **Conclusions:** Moral resilience was independently associated with both lower supervisory gaslighting and lower quiet quitting. These cross-sectional findings cannot establish causality. If confirmed in longitudinal research, interventions that strengthen moral resilience and improve supervisory practices may support employee engagement and wellbeing in white-collar workforces.

## 1. Introduction

Governmental organizations operate within complex regulatory and hierarchical structures. Employees face accountability pressures, strict procedures, and limited room for discretion. Conflict is a routine feature of this environment, and the way it is managed shapes collaboration, wellbeing, and the quality of public services [1]. When conflict is handled poorly, or when employees feel unable to raise concerns, organizational silence can take hold and erode trust and commitment [2,3]. These dynamics have been documented in Greek governmental bodies [2]. In the period following the COVID-19 pandemic, moral pressure and strain on the public workforce have intensified, which makes these dynamics especially timely [4]. Understanding the psychosocial conditions of public-sector work is therefore both a practical and a scientific concern.

A central part of this context is the ethical climate of the organization. Ethical climate refers to the shared perceptions of what is considered right and how ethical issues should be handled [5]. It works as an informal normative frame that guides behavior, decision-making, and the management of conflict. Many studies have linked it to commitment, satisfaction, and other work outcomes [6]. A comprehensive review has since mapped its antecedents and outcomes across many settings [7]. Ethical leadership reinforces this frame and is associated with stronger engagement and lower misconduct [8,9]. In the Greek public sector, a weak ethical climate has been associated with reluctance to speak up and with tolerance of wrongdoing [10]. Ethical climate and conflict management thus form the broader backdrop against which moral pressure, manipulation, and disengagement arise.

One increasingly visible form of disengagement is quiet quitting. The concept builds on long-standing work on personal engagement and disengagement at work [11] and on the measurement of work engagement [12]. Quiet quitting describes employees who meet the minimum requirements of their role but withdraw discretionary effort and emotional investment [13]. It is not the same as resignation or absenteeism. It is a quieter, more persistent withdrawal that can erode performance and morale, and it is closely tied to burnout and dissatisfaction [14]. It can also be interpreted as a self-protective mechanism, triggered when sustained demands do not have matching resources, thus pushing workers toward withdrawal. It has been described across disciplines and flagged as a risk for healthcare systems worldwide [15,16,17,18], and it appears common among public-service and healthcare workers [19]. Work engagement, and its effects on performance and wellbeing, also differs between the public and private sectors [20]. In governmental organizations, where motivation often depends on meaning and service rather than financial reward, quiet quitting may be particularly relevant.

A second phenomenon is workplace gaslighting. Gaslighting is a pattern of psychological manipulation in which a person is led to doubt their own judgment, memory, or perception of reality [21]. It has also been analyzed as a sociological phenomenon embedded in power relations and stereotypes, rather than as a purely individual one [22]. As an evolving concept, gaslighting overlaps with concepts such as emotional and verbal abuse, narcissistic behavior or workplace bullying, and this often blurs the line between abuse and everyday disagreement. The distinctive characteristic of gaslighting is the effort to change one’s perception of reality. At work, it usually involves a supervisor who undermines an employee’s confidence, denies stated facts, or distorts events [23]. Unlike open conflict, gaslighting is subtle and cumulative, and it is linked to anxiety, reduced self-trust, and withdrawal [23]. A scale to measure it in employees was recently developed and validated in the Greek working population [24]. Independent measures of gaslighting at work have also been developed in other countries [25]. Early evidence links workplace gaslighting to burnout and turnover intention [26], but its organizational correlates remain understudied.

A third construct is moral resilience. Rushton introduced the concept to describe the capacity to preserve integrity and function in the face of moral adversity [27,28]. Moral resilience includes moral courage, moral efficacy, self-regulation, and the ability to respond to ethically difficult situations without losing a sense of purpose [29]. It is measured with the Rushton Moral Resilience Scale, whose revised 16-item version shows sound psychometric properties [30,31] and has been validated in Greek [32]. Higher moral resilience has been associated with lower burnout, lower turnover intention, and lower quiet quitting [33]. Within the job demands and resources framework, moral resilience can be understood as a personal resource [34,35]. Although most research comes from healthcare, the construct is not profession-specific.

These three constructs do not operate in isolation, yet they have rarely been examined together, and evidence from outside healthcare is scarce. Most studies come from healthcare and focus on a single construct at a time [26,36]. The present study addressed this gap. It examined the levels of moral resilience, workplace gaslighting, and quiet quitting among employees of a Greek governmental organization with audit and payment responsibilities. It also examined how moral resilience relates to gaslighting and quiet quitting, and whether these relations differ by demographic and work-related characteristics. We tested two main expectations: first, that higher moral resilience would be associated with lower quiet quitting; and second, that higher moral resilience would be associated with lower gaslighting. By studying these relationships together, the study aimed to clarify how moral resilience relates to gaslighting and quiet quitting in a white-collar work setting.

**Figure 1:**
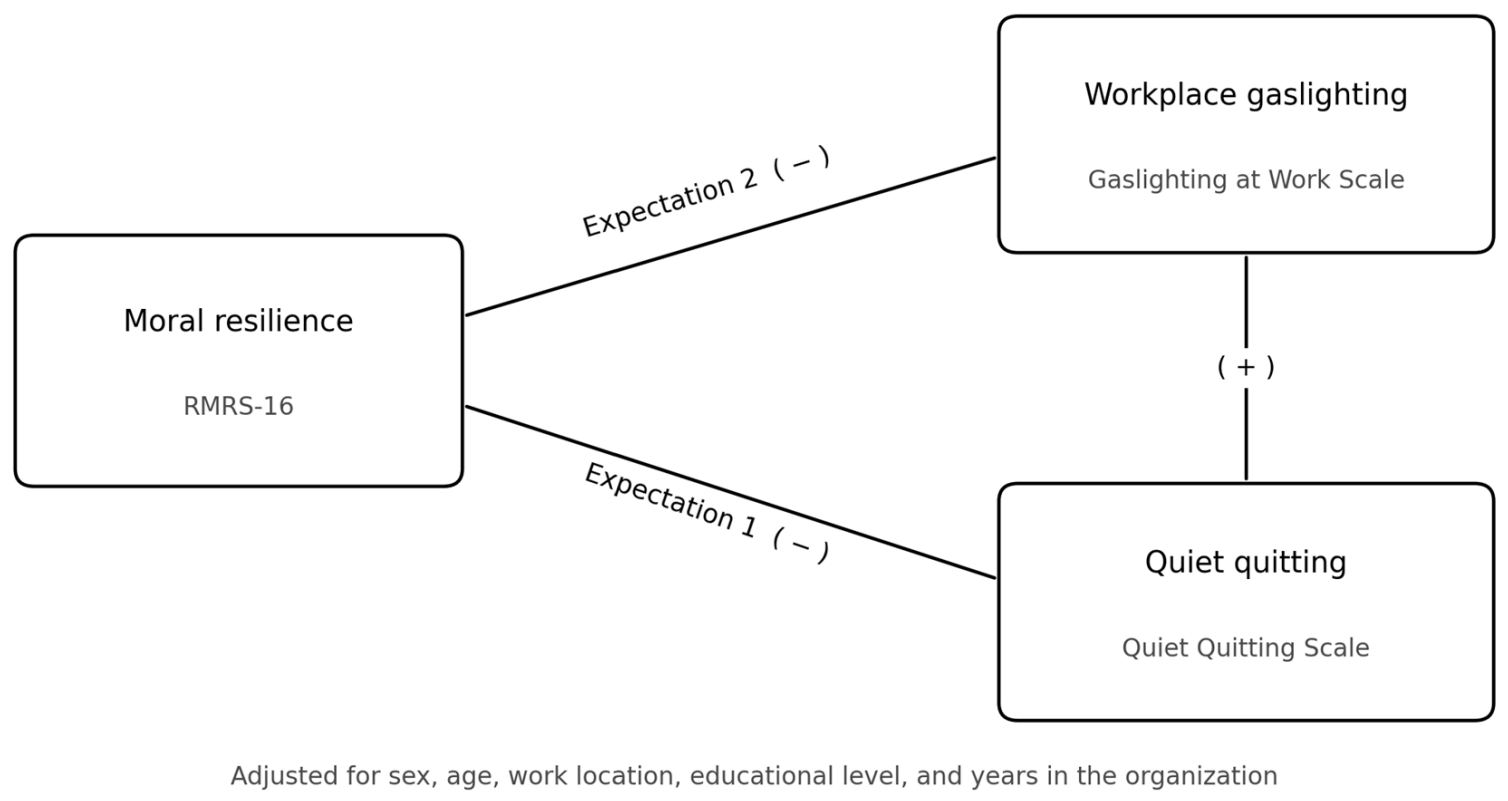
Associations examined in the study. The lines represent expected associations and not causal paths. Expectation 1 concerns moral resilience and quiet quitting. Expectation 2 concerns moral resilience and gaslighting. The signs indicate the expected direction of each association.

Figure 1 summarizes the associations that were examined.

## 2. Materials and Methods

### 2.1. Study Design

This was a cross-sectional, correlational study based on a self-report questionnaire. Data were collected at a single time point. The design fits the aim of the study, which was to map current attitudes and experiences rather than to track changes over time. For the gaslighting measure, participants were asked to report on the previous six months. The cross-sectional nature allows the description of associations but does not support causal inference [37].

### 2.2. Setting and Participants

The study took place in a large public organization in Greece with audit and payment responsibilities. Its staff are white-collar employees engaged in administrative, financial, and audit work. The organization operates through a central service and several regional directorates. At the time of the study, it employed 480 staff.

Inclusion criteria were an active employment relationship with the organization during the data collection period, at least six months of service, and voluntary, anonymous participation. The minimum service requirement ensured that responses reflected real and sustained experience of the work environment.

Questionnaires were distributed across directorates, departments, and offices in proportion to their size, so that the sample would reflect the structure of the organization. The final sample comprised 433 employees, which corresponds to about 90% of the target population. A sample of at least 10 to 15 participants per variable is considered adequate for correlational and multivariable analyses in quantitative organizational research [38]. The achieved sample met this requirement.

### 2.3. Measures

The questionnaire had two parts. The first part recorded demographic and work-related characteristics, including sex, age, educational level, work location, total years of work experience, and years of service in the organization. The second part consisted of three validated self-report scales.

#### 2.3.1. Quiet Quitting

Quiet quitting was measured with the Quiet Quitting Scale [13]. The scale has nine items rated on five-point scales. Seven items are rated on an agreement format (1 = strongly disagree; 2 = disagree; 3 = neither agree nor disagree; 4 = agree; 5 = strongly agree), and two items are rated on a frequency format (1 = never; 2 = rarely; 3 = sometimes; 4 = often; 5 = always). It captures three components: detachment, lack of initiative, and lack of motivation. Three positively worded items are reverse-scored, so that higher scores indicate stronger quiet quitting. Total scores are calculated as the mean of the item responses and range from 1 to 5. The scale was developed and validated in the Greek working population [13].

#### 2.3.2. Workplace Gaslighting

Workplace gaslighting was measured with the Gaslighting at Work Scale [24]. The scale has 11 items rated on a five-point frequency scale (1 = never; 2 = rarely; 3 = sometimes; 4 = very often; 5 = always). Participants reported on the behavior of their supervisor over the previous six months. The scale captures two components: loss of self-trust and abuse of power. Higher scores indicate more frequent exposure to gaslighting. Total scores are calculated as the mean of the item responses and range from 1 to 5. The scale was developed and validated in the Greek working population [24].

#### 2.3.3. Moral Resilience

Moral resilience was measured with the revised Rushton Moral Resilience Scale, RMRS-16 [31]. The scale has 16 items rated on a four-point agreement scale (1 = disagree; 2 = somewhat disagree; 3 = somewhat agree; 4 = agree). It captures four components: response to moral adversity, personal integrity, relational integrity, and moral efficacy. The Greek version was produced through a forward and backward translation procedure and validated in a Greek sample [32]. Negatively worded items are reverse-scored, so that higher scores indicate greater moral resilience. Total scores are calculated as the mean of the item responses and range from 1 to 4. The Greek version of the RMRS-16 was used exactly as validated, without any modification of item content, response format, or instructions.

### 2.4. Data Collection

Data were collected from 12 November to 12 December 2025. The questionnaire was self-administered and required about ten minutes to complete. It was distributed electronically to employees. No phase of data collection preceded the institutional and ethical approvals.

### 2.5. Ethical Considerations

Participation was voluntary. It was not linked to any service obligation, reward, or pressure. Participants received an information note describing the purpose of the study, the anonymity of responses, and the use of data exclusively for research purposes. Consent was implied through the free choice to complete the questionnaire, a practice consistent with guidance for minimal-risk research [39].

No identifying data were collected. Responses were analyzed and reported in aggregate form. Data were stored in a secure electronic environment, accessed only by the researcher, and processed in line with the General Data Protection Regulation [40].

### 2.6. Statistical Analysis

Categorical variables are presented as absolute and relative frequencies. Continuous variables are presented as mean, standard deviation, median, minimum, and maximum. The normality of continuous variables was assessed with the Kolmogorov–Smirnov test and normality plots. The study was reported in line with the STROBE guidelines for observational research [41].

The demographic and work-related characteristics were treated as independent variables throughout. Quiet quitting and gaslighting served as the dependent variables in the multivariable models, and moral resilience was entered as an independent variable in both. All analyses were conducted on complete cases, with listwise exclusion of any missing values from the relevant analysis. Total work experience and years of service in the organization were strongly correlated (Pearson r = 0.60, *p* < 0.001). To avoid multicollinearity, total work experience was not entered in the multivariable models.

The *t*-test was used for the relationship between a continuous variable and a dichotomous variable. The Pearson correlation coefficient was used for the relationship between two continuous variables. The Spearman correlation coefficient was used for the relationship between a continuous variable and an ordinal variable. Then we constructed two multivariable linear regression models. First, we constructed a multivariable model with quiet quitting as the dependent variable. In that case, we considered sex, age, work location, educational level, years in the organization, gaslighting and moral resilience as the independent variables. Second, we constructed a multivariable model with gaslighting as the dependent variable. In that case, we considered sex, age, work location, educational level, years in the organization and moral resilience as the independent variables. In both models all independent variables were entered simultaneously and were retained regardless of statistical significance. We did not apply stepwise selection procedures. The independent variables were chosen in advance as demographic and work-related characteristics that could plausibly relate to both the exposure and the outcomes. No variable was selected on the basis of the data. For each model we report the coefficient of determination as an indication of model fit. For the final models, the b coefficients, their 95% confidence intervals, and the *p*-values are reported. Multicollinearity was assessed through the calculation of variance inflation factors (VIFs), with values exceeding 4 considered indicative of potential collinearity issues. To evaluate multivariable normality, we inspected histograms of the residuals. Scatterplots of residuals against predicted values were also reviewed to assess homoscedasticity and linearity. The two-sided level of statistical significance was set at 0.05. Analyses were performed with IBM SPSS Statistics 28.0.

Several procedural steps were taken to limit common method bias. Participation was anonymous and voluntary, and respondents were assured that there were no right or wrong answers. The three constructs were measured with validated instruments that used different response formats and reference points [42]. Harman’s single-factor test was applied as a further statistical check. All scale items were entered into an unrotated factor analysis, and the variance explained by the first factor was examined [42].

To examine the measurement validity of the Quiet Quitting Scale, the Gaslighting at Work Scale, and the Rushton Moral Resilience Scale we performed confirmatory factor analysis (CFA). We calculated the Root Mean Square Error of Approximation (RMSEA), the Standardized Root Mean Square Residual (SRMR), the Comparative Fit Index (CFI), and the Tucker–Lewis Index (TLI). Values below 0.10 for RMSEA, below 0.08 for SRMR, and above 0.90 for CFI and TLI were considered acceptable. The CFA was performed with IBM SPSS Amos version 21 (IBM Corp., Armonk, NY, USA).

## 3. Results

### 3.1. Sample Characteristics

A total of 433 employees took part, which corresponds to about 90% of the target population. Most participants were women (77.8%). The mean age was 51.4 years (SD 6.2), with a range from 30 to 65 years. Half of the sample worked in Athens (51.0%), while the rest were spread across regional services. Most employees held a higher education degree (86.1%), and about half also held a postgraduate qualification (48.1%). The mean total work experience was 24.9 years (SD 6.7). The mean length of service in the organization was 19.5 years (SD 7.1). Table 1 presents the full profile.

### 3.2. Reliability and Descriptive Statistics

Internal consistency was good to excellent for all three instruments. Cronbach’s alpha was 0.808 for the Quiet Quitting Scale, 0.911 for the Gaslighting at Work Scale, and 0.807 for the RMRS-16. The mean quiet quitting score was 2.26. The mean gaslighting score was 1.84. The mean moral resilience score was 3.08. Among the quiet quitting components, lack of motivation showed the highest mean (2.88) and detachment the lowest (1.94). For gaslighting, abuse of power (2.14) was reported more often than loss of self-trust (1.60). Table 2 presents the reliability coefficients and descriptive statistics for each scale and its subscales. Item-level descriptive statistics for the three scales are provided in Supplementary Table S1.

### 3.3. Measurement Validity

Results from CFA for the Quiet Quitting Scale, the Gaslighting at Work Scale, and the Rushton Moral Resilience Scale are shown in Table 3. Measurement validity was acceptable for all three scales. RMSEA values ranged from 0.058 to 0.093 and SRMR values from 0.035 to 0.048. CFI values ranged from 0.952 to 0.968 and TLI values from 0.922 to 0.942. The RMSEA for the Gaslighting at Work Scale (0.093) approached the 0.10 acceptability threshold and should be regarded as borderline, although the SRMR, CFI, and TLI values for this scale were well within the acceptable ranges. The first unrotated factor accounted for 22.2% of the total variance, which is below the 50% threshold. Common method bias was therefore unlikely to have a substantial effect on the results.

### 3.4. Bivariate Associations with Quiet Quitting

Bivariate associations with quiet quitting are presented in Table 4. Men reported higher quiet quitting than women (*p* < 0.001). Younger employees reported higher quiet quitting (r = −0.21, *p* < 0.001). Higher moral resilience was associated with lower quiet quitting (r = −0.37, *p* < 0.001). Educational level, work location, and years of service in the organization showed no significant association.

### 3.5. Bivariate Associations with Gaslighting

Bivariate associations with gaslighting are presented in Table 5. Men reported more frequent gaslighting than women (*p* = 0.02). Younger employees reported more gaslighting (r = −0.14, *p* < 0.01). Higher moral resilience was associated with less gaslighting (r = −0.27, *p* < 0.001). Educational level, work location, and years of service in the organization showed no significant association.

### 3.6. Factors Associated with Quiet Quitting

Multivariable linear regression with quiet quitting as the dependent variable is shown in Table 6. Men reported higher quiet quitting than women. Younger employees reported higher quiet quitting. Higher gaslighting was associated with higher quiet quitting. Higher moral resilience was associated with lower quiet quitting. The model explained 32% of the variance in quiet quitting. All variance inflation factors were below 2, and residual inspection showed no material violation of the model assumptions (Supplementary Figures S1 and S2). The same pattern recurred across the quiet quitting subscales, where higher gaslighting and lower moral resilience were consistently associated with greater detachment, lack of initiative, and lack of motivation.

### 3.7. Factors Associated with Gaslighting

Multivariable linear regression with gaslighting as the dependent variable is shown in Table 7. Men reported higher gaslighting than women. Younger employees reported higher gaslighting. Employees with a lower educational level reported higher gaslighting. We found a positive association between years in the organization and levels of gaslighting. Higher moral resilience was associated with lower gaslighting. The model explained 16.1% of the variance in gaslighting. All variance inflation factors were below 2, and residual inspection showed no material violation of the model assumptions (Supplementary Figures S3 and S4).

## 4. Discussion

This study examined moral resilience, workplace gaslighting, and quiet quitting among employees of a Greek governmental organization. Three findings stand out. Quiet quitting behaviors were reported across this workforce. Higher moral resilience was associated with lower quiet quitting. Higher moral resilience was also associated with lower gaslighting. Taken together, the results are consistent with a protective role of moral resilience in a demanding white-collar setting, although the design does not allow causal claims.

The mean quiet quitting score sat below the midpoint of the scale, but the lack of motivation component was well above the other two. This points to real withdrawal in a workforce with long tenure and stable employment, and it suggests that disengagement here is not driven by job insecurity. It is more likely a response to the work environment itself. Governmental organizations often combine heavy procedural demands with limited autonomy and slow recognition. Such conditions can lead employees to protect themselves by doing what is required and no more. This reading is consistent with accounts that treat quiet quitting as a reaction to organizational conditions rather than a personal failing [13]. Comparable levels of quiet quitting have been documented among employees in other European settings [43]. It also fits the job demands and resources perspective, in which sustained demands without matching resources push workers toward withdrawal [34,44].

Moral resilience showed consistent negative associations with both outcomes. In the regression for quiet quitting, moral resilience and gaslighting were the two strongest correlates. They were comparable in magnitude and far larger than any demographic variable, with moral resilience in the opposite direction. This pattern is consistent with a protective role, although the direction of the association cannot be established. Employees who could preserve their integrity and sense of purpose under moral pressure were less likely to withdraw. This is in line with work that links moral resilience to lower burnout, lower turnover intention, and better wellbeing [33,45]. The result extends evidence beyond healthcare, where most studies have been conducted, into a white-collar, administrative workforce. It also aligns with international findings. Among Chinese nurses, higher moral resilience was associated with lower turnover intention through reduced burnout [46], and the scale has been adapted and validated across cultures [47]. Studies conducted during the COVID-19 pandemic linked moral resilience to better mental health under sustained moral pressure [4,48]. The pattern we observed echoes a recent study in a Greek nursing sample, where higher resilience was associated with less gaslighting and less quiet quitting [49]. Our findings extend this to moral resilience specifically and to a white-collar workforce outside healthcare. They also fit the wider evidence that moral distress, the strain that moral resilience helps to withstand, is consistently linked to emotional exhaustion in health workers [50].

The link between moral resilience and gaslighting deserves attention. Higher moral resilience was associated with lower reported gaslighting. The direction of this relationship cannot be settled by a cross-sectional design. Resilient employees may interpret and resist manipulative behavior differently, so they report less of it. It is also possible that sustained exposure to gaslighting wears down moral resilience over time. Both readings are compatible with the data. A third possibility is reporting bias. Employees with higher moral resilience may be less inclined to label ambiguous supervisory behavior as gaslighting. Part of the association could therefore reflect how such behavior is perceived and reported rather than a difference in actual exposure. The self-report nature of the gaslighting measure cannot rule this out. Whichever reading applies, the two constructs are connected, and the connection matters for how employees experience authority and conflict at work.

Gaslighting was also independently associated with quiet quitting. Employees who reported more frequent manipulative behavior from their supervisor reported more withdrawal, and the association held after adjustment for moral resilience and for demographic characteristics. This is in line with Greek evidence connecting workplace gaslighting to burnout, turnover intention, and poorer mental health at work [26,36]. One reading is that gaslighting strips away the resources that sustain discretionary effort. When an employee’s account of events is repeatedly questioned, raising concerns stops feeling safe, and minimal compliance becomes the lower-risk option. The design cannot separate this from the reverse path, in which already disengaged employees read supervisory behavior more negatively.

Demographic patterns were modest but consistent. Men reported higher quiet quitting and higher gaslighting than women. Younger employees reported more of both. Lower educational levels, and longer service in the organization, were associated with higher reported gaslighting in the adjusted model. Neither showed an association at the bivariate level, so both should be read as tentative. The direction of the sex difference is worth noting. Much of the gaslighting literature describes women as the more frequent targets, in line with accounts that root the phenomenon in gendered power relations [22]. Several explanations are possible for the opposite pattern seen here. Men were a minority of this workforce (22.2%), the measure was based on self-report, and the associations were small. Men and women may also differ in how they perceive or are willing to report manipulation and disengagement. These results should therefore be read with caution and not as evidence that women experience less gaslighting in general. Taken together, the small demographic effects suggest that disengagement and manipulation are shaped more by the work environment than by who the employee is.

The size of the observed associations also deserves comment. The bivariate correlations of age and moral resilience with the two outcomes ranged from 0.14 to 0.37 in absolute value, which corresponds to small to moderate effects. Associations of this magnitude are typical in organizational research and do not support strong prediction at the level of the individual employee. Their practical relevance lies at the group level. Even modest associations can translate into meaningful differences across a workforce, particularly when they involve a modifiable resource such as moral resilience. The regression findings should be read in the same spirit, as consistent signals rather than precise forecasting tools.

The broader backdrop is the ethical climate of the organization and the way conflict is handled. Neither construct was measured here, so no empirical link can be drawn, and both are noted only as context for interpreting supervisory behavior. In the literature, weak ethical climates and poorly managed conflict have been connected with manipulation, organizational silence, and disengagement [5,8,51]. Employees who feel able to speak without fear tend to stay engaged [52]. Moral resilience may help individuals withstand a difficult climate, but it cannot replace healthy organizational conditions.

These patterns are best read against the international picture. Global workforce surveys show that only a minority of employees are actively engaged, and the engaged share is lowest in Europe and higher in the United States and Canada [53]. Employees who are present but psychologically detached are described in those surveys as quietly quitting, which places the high level seen here within a broader European pattern of low engagement. Work engagement and its drivers also vary across sectors and countries. Employees in administrative roles often draw on intrinsic, service-oriented motivation rather than material reward, yet they work within procedural constraints and limited autonomy [20,54]. The other two constructs have a comparable cross-national footprint. Evidence on undermining and abusive supervision comes mostly from the United States [55,56], and the sociological account of gaslighting is also largely American [22]. Evidence on moral resilience as a protective resource now reaches beyond the United States to China. There it has been linked to lower turnover intention, and the scale has been adapted for local use [46,47]. Greece, and Europe more broadly, remains comparatively understudied, which is part of what motivated this study.

### 4.1. Practical and Policy Implications

The following implications are potential rather than evidence-based recommendations, since the cross-sectional design cannot show that changing one variable would change another. With this caveat, the findings suggest a dual approach for organizations of this kind that would be worth testing, addressing both organizational conditions and individual resources.

- Organizational level. Organizations could strengthen supervision and the ethical work environment. Structured training for supervisors in ethical leadership, constructive conflict management, and the recognition of manipulative behavior may reduce exposure at its source [1,57]. Clear policies on undermining conduct, supported by confidential reporting channels, could give employees a safe route to raise concerns. Regular assessment of the ethical climate could help sustain a healthier environment. Structured conflict-resolution mechanisms, such as mediation, may prevent disagreements from escalating into manipulative behavior. Organization-wide civility initiatives have improved interpersonal climate and engagement in large public health systems and offer a tested model for such efforts [58].
- Individual level. Organizations could invest in building moral resilience. Experiential programs that combine reflection, mindfulness, and skills practice have improved moral resilience, ethical confidence, and work engagement among health workers. They have also reduced burnout and turnover intention [59,60]. Workshops on moral courage, emotional regulation under pressure, and ethical decision-making could be integrated into existing staff development. Mentoring or peer-support programs may be especially valuable for younger employees, who appeared more vulnerable in this study.

Given the levels of quiet quitting observed here, even modest improvements in moral resilience and supervisory practice could yield meaningful benefits for employee wellbeing and the quality of public services. A practical starting point would be a pilot program in moral resilience and ethical leadership for managers and staff, evaluated before and after. Quiet quitting could also be monitored regularly using the validated scale. Intervention studies are needed to test whether such benefits materialize. It is then up to upper management to decide whether this pilot program could be expanded to cover more public organizations or prioritize between them based on each department’s staff needs.

### 4.2. Theoretical Contributions

This study contributes to existing literature in three ways. First, it is among the first to examine moral resilience, gaslighting, and quiet quitting together in a non-healthcare, white-collar context. Second, it provides empirical support for moral resilience as a personal resource within the job demands and resources model in bureaucratic environments. Third, it documents the association between supervisory behavior and employee disengagement in a Greek white-collar workforce.

### 4.3. Strengths and Limitations

Several limitations should be noted. The design was cross-sectional, so causal claims are not possible, and the role of moral resilience can be described only as an association. The data were self-reported. Procedural steps were taken to limit common method bias, including anonymity and the use of instruments with different response formats. Harman’s single-factor test also indicated that common method bias was unlikely to have a substantial effect on the results [42]. That test is a relatively insensitive check, however, and self-report bias cannot be fully excluded.

A key limitation is that the study was conducted in a single governmental organization with a relatively homogeneous workforce, which was predominantly female, middle-aged, and long-serving. Despite the large sample and the high response rate of about 90%, this restricts how far the findings can be generalized. Those who chose to take part may also differ from those who did not. Accordingly, the conclusions should be read as specific to this organization. Replication across multiple organizations, occupational sectors, and cultural settings is needed before broader generalization is warranted. The broad constructs of ethical climate and conflict management were treated as context and were not measured directly. Published cut-off scores exist for the quiet quitting and gaslighting scales [61,62], but they were derived in other occupational samples, and their transferability to this population is uncertain. For this reason, no prevalence estimates based on these thresholds are reported, and all analyses rely on the continuous scale scores.

The relatively low explanatory power of the gaslighting model (R^2^ = 16.1%) indicates that workplace gaslighting is explained predominantly by factors not captured in the present analysis. Gaslighting is an interpersonal and organizational phenomenon. Workplace characteristics such as managerial behavior, organizational justice, toxic leadership, power asymmetries, and support from supervisors and colleagues may be more strongly associated with it than individual sociodemographic characteristics. Moral resilience should therefore not be read as a primary determinant of workplace gaslighting, but as an individual-level factor that operates within a broader organizational context. The association between higher moral resilience and lower gaslighting persisted after adjustment, which suggests an independent association. The effect size should still be read in light of the substantial unexplained variance. The study also did not examine the mechanisms underlying the association between moral resilience and quiet quitting. Both variables were included in the same multivariable model, but we did not test whether gaslighting mediates or moderates that association. Employees with higher moral resilience may be less exposed to gaslighting, or less affected by it, which could in turn reduce disengagement. Future studies should employ mediation and moderation analyses, preferably with longitudinal designs.

The interpretation of sex differences also calls for caution. Male participants reported higher levels of workplace gaslighting and quiet quitting, but the sample was predominantly female and the subgroup of men was small. Estimates for the sex coefficients may therefore be less precise and more open to sampling variability. Replication in samples with a more balanced sex distribution is warranted. A further limitation is that we did not account for potential clustering of gaslighting experiences within departments or work units. Employees in the same department may be exposed to similar managerial practices, organizational norms, and interpersonal dynamics, which can produce correlated responses. Department-level information was not recorded, so multilevel analyses could not be performed. Future studies should incorporate organizational structure variables and hierarchical modeling. The strengths include the large sample, the use of validated instruments, and reporting in line with the STROBE guidelines [41]. These support the internal consistency of the findings within this organization.

## 5. Conclusions

This study examined moral resilience, workplace gaslighting, and quiet quitting among employees of a Greek governmental organization. Quiet quitting behaviors were reported across the workforce, and they were linked to the experience of gaslighting from supervisors. Moral resilience stood out as the factor most consistently associated with lower disengagement. This pattern is consistent with a protective role, but the cross-sectional design cannot confirm it.

The results suggest that disengagement in this setting reflects working conditions more than personal characteristics. Manipulative supervisory behavior and low moral resilience were the factors most consistently associated with withdrawal. Demographic variables played only a minor role.

These findings are relevant for administrative and white-collar workforces. Reducing quiet quitting calls for attention to the quality of supervision and to the presence of undermining behavior. Strengthening moral resilience, through training, ethical leadership, and clear standards, may help employees withstand pressure without disengaging. Such measures cannot substitute for a healthy ethical climate, but they can reinforce it.

Future research should follow these relationships over time, across several organizations, and with direct measures of ethical climate and conflict management. Work of this kind would clarify how moral resilience, gaslighting, and quiet quitting shape one another. An important research question would be whether prolonged exposure to gaslighting gradually diminishes moral resilience over time. Another practical question would concern the ways in which quiet quitting can be more effectively reversed. Evidence of this kind is necessary if governmental organizations aspire to protect the wellbeing and engagement of their staff.

## Figures and Tables

**Table 1:** Demographic and work-related characteristics of the participants (*n* = 433).

Characteristic	*n*	%
Sex		
Men	96	22.2
Women	337	77.8
Age, years (mean, SD)	51.4	6.2
Work location		
Athens	221	51.0
Thessaloniki	16	3.7
Larisa	20	4.6
Patra	8	1.8
Crete	20	4.6
Other city	148	34.2
Educational level		
Secondary (high school)	48	11.1
Technical school	12	2.8
Technological institute	49	11.3
University	116	26.8
Master’s degree	196	45.3
Doctoral degree	12	2.8
Total work experience, years (mean, SD)	24.9	6.7
Service in the organization, years (mean, SD)	19.5	7.1

**Table 2:** Internal consistency and descriptive statistics of the study instruments.

Scale/Subscale	Cronbach’s Alpha	Mean	SD
Quiet quitting (total)	0.808	2.26	0.69
Detachment	0.717	1.94	0.69
Lack of initiative	0.723	2.23	0.78
Lack of motivation	0.873	2.88	0.99
Gaslighting (total)	0.911	1.84	0.76
Loss of self-trust	0.872	1.60	0.76
Abuse of power	0.927	2.14	0.88
Moral resilience (total)	0.807	3.08	0.43
Response to moral adversity	0.829	2.44	0.84
Personal integrity	0.712	3.76	0.36
Relational integrity	0.728	2.81	0.65
Moral efficacy	0.708	3.33	0.47

**Table 3:** Confirmatory factor analysis for the Quiet Quitting Scale, the Gaslighting at Work Scale, and the Rushton Moral Resilience Scale.

Scale	RMSEA	SRMR	CFI	TLI
Quiet Quitting Scale	0.080	0.046	0.960	0.927
Gaslighting at Work Scale	0.093	0.035	0.968	0.942
Rushton Moral Resilience Scale	0.058	0.048	0.952	0.922

RMSEA: Root Mean Square Error of Approximation; SRMR: Standardized Root Mean Square Residual; CFI: Comparative Fit Index; TLI: Tucker–Lewis Index.

**Table 4:** Bivariate associations between participant characteristics, moral resilience, and quiet quitting (*n* = 433).

Characteristic	Mean (SD)	r	*p*
Sex ^a^			<0.001
Men	2.48 (0.70)		
Women	2.21 (0.67)		
Age ^b^		−0.21	<0.001
Work location ^a^			0.86
Athens	2.26 (0.62)		
Other	2.26 (0.74)		
Educational level ^c^		0.09	0.06
Years in the organization ^b^		−0.05	0.35
Moral resilience ^b^		−0.37	<0.001

^a^ Independent-samples *t*-test (group means and standard deviations shown). ^b^ Pearson correlation. ^c^ Spearman correlation.

**Table 5:** Bivariate associations between participant characteristics, moral resilience, and gaslighting (*n* = 433).

Characteristic	Mean (SD)	r	*p*
Sex ^a^			0.02
Men	2.00 (0.89)		
Women	1.79 (0.72)		
Age ^b^		−0.14	0.004
Work location ^a^			0.38
Athens	1.87 (0.76)		
Other	1.81 (0.76)		
Educational level ^c^		−0.06	0.19
Years in the organization ^b^		0.04	0.42
Moral resilience ^b^		−0.27	<0.001

^a^ Independent-samples *t*-test (group means and standard deviations shown). ^b^ Pearson correlation. ^c^ Spearman correlation.

**Table 6:** Multivariable linear regression with quiet quitting as the dependent variable.

Independent Variable	b	95% Confidence Interval	*p*	Variance Inflation Factor
Men vs. women	0.32	0.18 to 0.45	<0.001	1.153
Age	−0.03	−0.04 to −0.01	<0.001	1.756
Work location (Athens vs. other)	0.06	−0.05 to 0.17	0.297	1.120
Educational level	0.04	−0.003 to 0.08	0.070	1.164
Years in the organization	0.01	−0.002 to 0.02	0.105	1.486
Gaslighting score	0.27	0.19 to 0.35	<0.001	1.200
Moral resilience score	−0.52	−0.65 to −0.39	<0.001	1.100

The model explained 32% of the variance in quiet quitting.

**Table 7:** Multivariable linear regression with gaslighting as the dependent variable.

Independent Variable	b	95% Confidence Interval	*p*	Variance Inflation Factor
Men vs. women	0.36	0.20 to 0.53	<0.001	1.108
Age	−0.04	−0.05 to −0.03	<0.001	1.623
Work location (Athens vs. other)	0.11	−0.04 to 0.24	0.14	1.115
Educational level	−0.10	−0.15 to −0.05	<0.001	1.126
Years in the organization	0.02	0.01 to 0.03	<0.001	1.439
Moral resilience score	−0.49	−0.65 to −0.34	<0.001	1.011

The model explained 16.1% of the variance in gaslighting.

## Data Availability

The data presented in this study are available on request from the corresponding author. The data are not publicly available due to privacy restrictions.

## Supplementary Materials

The following supporting information can be downloaded at: https://www.mdpi.com/article/10.3390/healthcare14162552/s1, Table S1: Item-level and subscale-level descriptive statistics for the three study instruments; Figure S1: Histogram of the residuals with quiet quitting as the dependent variable; Figure S2: Scatterplot of residuals versus predicted values with quiet quitting as the dependent variable; Figure S3: Histogram of the residuals with gaslighting as the dependent variable; Figure S4: Scatterplot of residuals versus predicted values with gaslighting as the dependent variable.

## Abbreviations

CI, confidence interval; JD-R, job demands and resources; QQS, Quiet Quitting Scale; RMRS-16, revised 16-item Rushton Moral Resilience Scale; SD, standard deviation; STROBE, Strengthening the Reporting of Observational Studies in Epidemiology.
