# Supplementary material for: Moral Resilience and Its Associations with Workplace Gaslighting and Quiet Quitting: A Cross-Sectional Study of White-Collar Workers in Greece
Source: Healthcare (Basel). 2026 Aug 14;14(16):2552. doi: 10.3390/healthcare14162552 (PMC13511810; doi:10.3390/healthcare14162552)
Supplement: Supplementary file 1 [file healthcare-14-02552-s001.zip › healthcare-4411749-supplementary.pdf]

Supplementary Materials

Moral Resilience and Its Associations with Workplace Gaslighting and Quiet Quitting: A Cross-Sectional Study of White-Collar Workers in Greece

Table S1. Item-level and subscale-level descriptive statistics for the three study instruments (n = 433).

Item	Item content (brief description)	Mean	SD
	**Quiet Quitting Scale (total, 9 items)**	**2.26**	**0.69**
	Detachment (4 items)	*1.94*	*0.69*
Item 1	Doing only the basic or minimum amount of work, without going above and beyond	1.87	0.99
Item 2	Letting a colleague take over part of one's own work	2.32	1.06
Item 3	Taking as many breaks as possible	2.01	1.01
Item 4	Pretending to work in order to avoid another task	1.59	0.76
	Lack of initiative (3 items)	*2.23*	*0.78*
Item 5	Not expressing opinions and ideas for fear of being assigned more tasks	1.91	1.00
Item 6	Not expressing opinions and ideas because working conditions are not going to change	2.41	1.06
Item 7	Taking initiative at work (R)	2.36	0.93
	Lack of motivation (2 items)	*2.88*	*0.99*
Item 8	Finding motives in one's job (R)	2.82	1.05
Item 9	Feeling inspired when working (R)	2.94	1.06
	**Gaslighting at Work Scale (total, 11 items)**	**1.84**	**0.76**
	Loss of self-trust (5 items)	*1.60*	*0.76*
Item 1	The supervisor denies saying things that the employee remembers him/her saying	1.86	0.97
Item 2	The supervisor lies to the employee	1.88	1.01
Item 3	The supervisor tells the employee that he/she is "imagining" things	1.41	0.78
Item 4	The supervisor accuses the employee of lying when he/she disagrees	1.35	0.76
Item 5	The supervisor twists or misrepresents the employee's words or ideas	1.50	0.90
	Abuse of power (6 items)	*2.14*	*0.88*
Item 6	The supervisor makes the employee doubt his/her qualifications	1.64	0.93
Item 7	The supervisor ignores the employee's complaints and concerns	2.49	1.16
Item 8	The supervisor tries to manipulate the employee	1.90	1.09
Item 9	The supervisor makes the employee depend on him/her for decisions about the employee's work	1.91	1.12
Item 10	The supervisor does not give clear instructions about what to do	2.54	1.22
Item 11	The supervisor does not tolerate disagreement or criticism	2.36	1.18
	**Rushton Moral Resilience Scale (RMRS-16) (total, 16 items)**	**3.08**	**0.43**
	Response to moral adversity (4 items)	*2.44*	*0.84*
Item 3	Feeling powerless in difficult ethical situations (R)	2.56	1.05
Item 5	Feeling overwhelmed by persistent ethical conflicts (R)	2.52	1.03

Item	Item content (brief description)	Mean	SD
Item 7	Being weighed down by lingering distress after challenging ethical situations (R)	2.16	0.97
Item 9	Pushing oneself beyond healthy limits when confronted with an ethical challenge (R)	2.51	1.10
	Personal integrity (4 items)	*3.76*	*0.36*
Item 2	Acting consistently with one's values regardless of the situation	3.81	0.48
Item 4	Having the conviction to act in accordance with one's values	3.75	0.55
Item 6	Taking responsibility for one's choices	3.87	0.34
Item 8	Having the courage to take action when others resist	3.59	0.55
	Relational integrity (4 items)	*2.81*	*0.65*
Item 11	Being distracted by others' strong emotions when ethical conflicts occur (R)	2.27	0.86
Item 13	Compromising one's values when others criticize one's opinions (R)	2.71	1.16
Item 14	Avoiding conflict with those in authority rather than acting on one's values (R)	2.82	1.01
Item 16	Acting in a way that compromises one's values because of fear (R)	3.44	0.87
	Moral efficacy (4 items)	*3.33*	*0.47*
Item 1	Voicing ethical concerns in a way that others take seriously	3.10	0.82
Item 10	Being able to articulate the ethical conflict when confronted with a challenge	3.37	0.73
Item 12	Feeling confident in one's ability to reason through ethical challenges at work	3.62	0.63
Item 15	Thinking clearly when confronting an ethical challenge, even under pressure	3.24	0.82

Items are identified by their number in the original scale publications and are grouped by subscale; brief content descriptions are provided in place of the full item wording. The full wording of the Quiet Quitting Scale and the Gaslighting at Work Scale is available in the open-access validation publications (Galanis et al., 2023; Katsiroumpa et al., 2025), and the RMRS-16 brief descriptions are paraphrased from the official instrument (Rushton Moral Resilience Scale-16, © Johns Hopkins University; Rushton et al., 2024), whose full wording is available from the developers; the instrument is copyright-protected. For the Quiet Quitting Scale, seven items use an agreement format (1 = strongly disagree; 2 = disagree; 3 = neither agree nor disagree; 4 = agree; 5 = strongly agree) and two items (Items 4 and 7) use a frequency format (1 = never; 2 = rarely; 3 = sometimes; 4 = often; 5 = always). The Gaslighting at Work Scale is rated on a five-point frequency scale (1 = never; 2 = rarely; 3 = sometimes; 4 = very often; 5 = always), with respondents reporting the behavior of their supervisor over the previous six months. The RMRS-16 is rated on a four-point agreement scale (1 = disagree; 2 = somewhat disagree; 3 = somewhat agree; 4 = agree), and its item-to-subscale assignment and reverse-scored items follow the Greek validation of the scale (Katsiroumpa et al., 2024). Items marked (R) are reverse-scored; item-level statistics are reported after reverse-scoring, so that higher scores indicate higher quiet quitting, more frequent gaslighting, and greater moral resilience. Subscale and total-scale means and standard deviations correspond to those reported in Table 2 of the main text. Item-level statistics were computed on all available responses for each item, whereas scale and subscale scores were computed on complete cases at the participant level; values reconstructed by aggregating the item-level statistics may therefore differ slightly from the participant-level scores, and the participant-level values reported in Table 2 are authoritative.

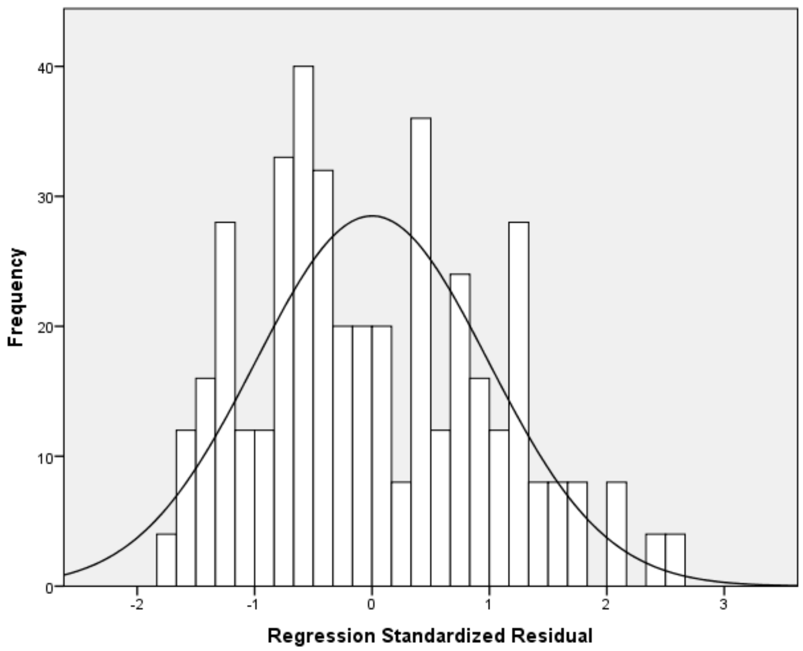

Figure S1. Histogram of the residuals with quiet quitting as the dependent variable.

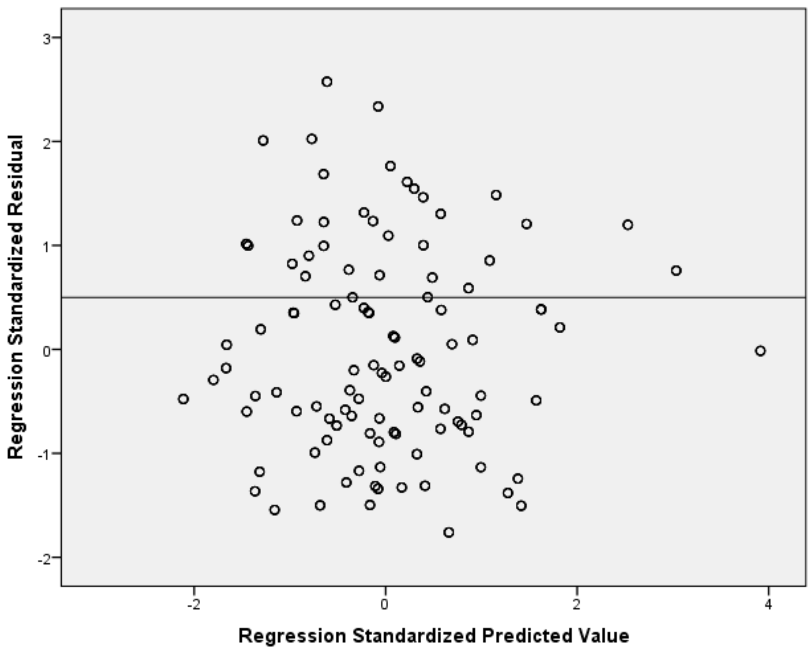

Figure S2. Scatterplot of residuals versus predicted values with quiet quitting as the dependent variable.

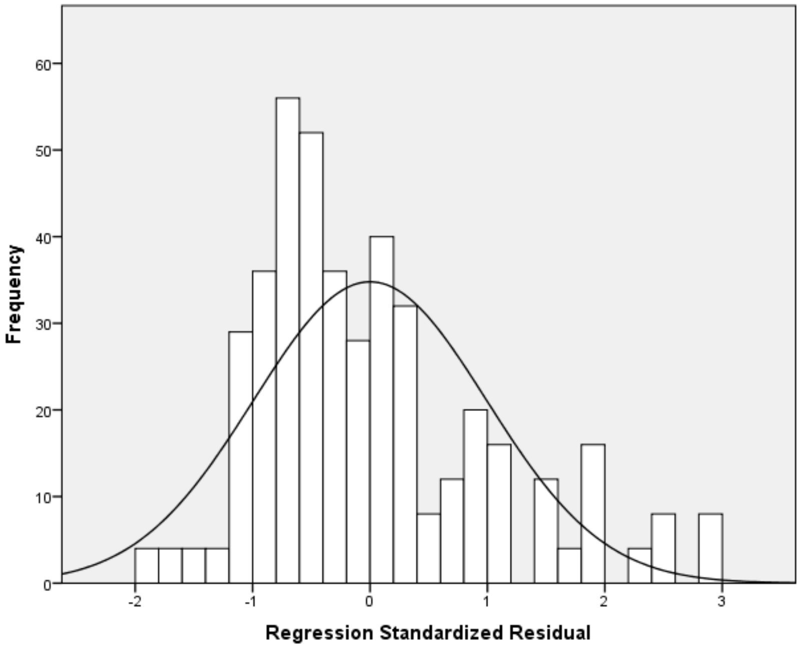

Figure S3. Histogram of the residuals with gaslighting as the dependent variable.

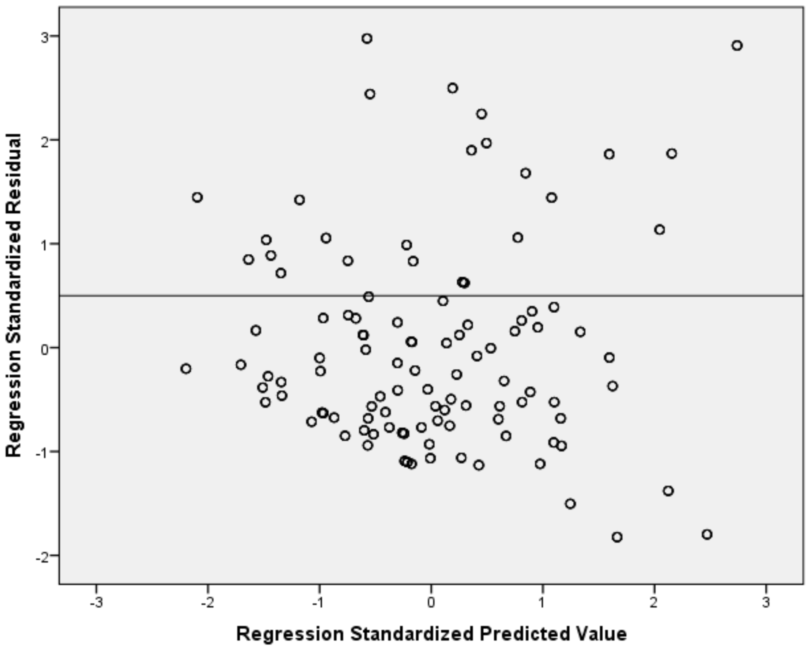

Figure S4. Scatterplot of residuals versus predicted values with gaslighting as the dependent variable.
